# Annular pancreas causing pyloric stenosis in a 66-year-old patient treated with gastrojejunostomy: a case report

**DOI:** 10.1093/jscr/rjab125

**Published:** 2021-04-22

**Authors:** P Patel, S Diko, S Kulkarni, C Persaud, F Sartorato, D Christian

**Affiliations:** 1 St. George’s University School of Medicine, Grenada, West Indies; 2 St. Joseph’s University Medical Center, Paterson, NJ 07503, USA

## Abstract

Annular pancreas is defined by a ring of pancreatic tissue encircling the descending portion of the duodenum. It is exceptionally rare in adults and commonly diagnosed during the investigation of symptoms arising due to its complications. Treatment usually involves the surgical correction with a duodenoduodenostomy, gastrojejunostomy or duodenojejunostomy. We discuss the case of a 66-year-old male patient who presented with symptoms of gastric outlet obstruction and was found to have an annular pancreas encircling the pylorus and the first and second portions of the duodenum and was treated by performing a gastrojejunostomy. Upper gastrointestinal series, computerized tomography (CT) scans, and magnetic resonance cholangeopancreatographys can all be used for preoperative diagnosis; however, endoscopic retrograde cholangiopancreatography (ERCP) is the diagnostic modality of choice. Nonetheless, many patients may only be diagnosed intraoperatively, especially those who cannot undergo an ERCP due to stenosis proximal to the duodenum or patients in whom the annulus may not be visible on CT scan.

## BACKGROUND

The pancreas forms when one dorsal and two ventral buds of the primitive foregut rapidly fuse due to expansion of the duodenum by the seventh week of gestation. Annular pancreas is formed when the ventral bud incompletely rotates [1]. It is characterized by a ring of pancreatic tissue most commonly surrounding the descending portion of the duodenum. There is a preponderance of cases of annular pancreas in the infant/newborn population. It is exceptionally rare in the adult population with incidences varying from 0.005% to 0.015%, and presentation is variable and can include duodenal obstruction, pancreatitis or symptoms of peptic ulcer disease [2, 3].

Many imaging studies can be used to diagnose annular pancreas. Upper gastrointestinal series may show an obstruction with proximal dilation. Computerized tomography (CT) scans may show a complete or partial ring of tissue surrounding the duodenum. Magnetic resonance cholangeopancreatography (MRCP) may show dilation of intrahepatic and/or extrahepatic bile ducts and possibly obstruction of bile flow (Figure 2). Endoscopic retrograde cholangiopancreatography (ERCP) is the diagnostic modality of choice and may show duodenal compression due to annular pancreas [4].

Treatment involves the surgical correction with a duodenoduodenostomy, a gastrojejunostomy or a duodenojejunostomy. These bypass procedures are preferred over annular resection due to the complications associated with surgery, including pancreatitis, pancreatic fistula formation and incomplete relief of obstruction, associated with annular resection [5]. Resection also results in a lower rate of permanent cure [6]. Gastrojejunostomy (with or without gastric resection) can be performed with additional vagotomy to prevent ulcerations.

We discuss the case of a 66-year-old male patient who presented with symptoms of gastric outlet obstruction who was found to have an annular pancreas encircling the pylorus and the first and second portions of the duodenum. This patient was challenging to diagnose because he had pyloric stenosis and could not undergo an ERCP to aid in preoperative diagnosis.

## CASE REPORT

The patient is a 66-year-old male with a past medical history of stroke, hypertension, hyperlipidemia and gastric ulcers (diagnosed in the Dominican Republic) who presented to the emergency department with complaints of upper abdominal pain, decreased appetite, weight loss and nausea exacerbated by oral intake.

**Figure 1:**
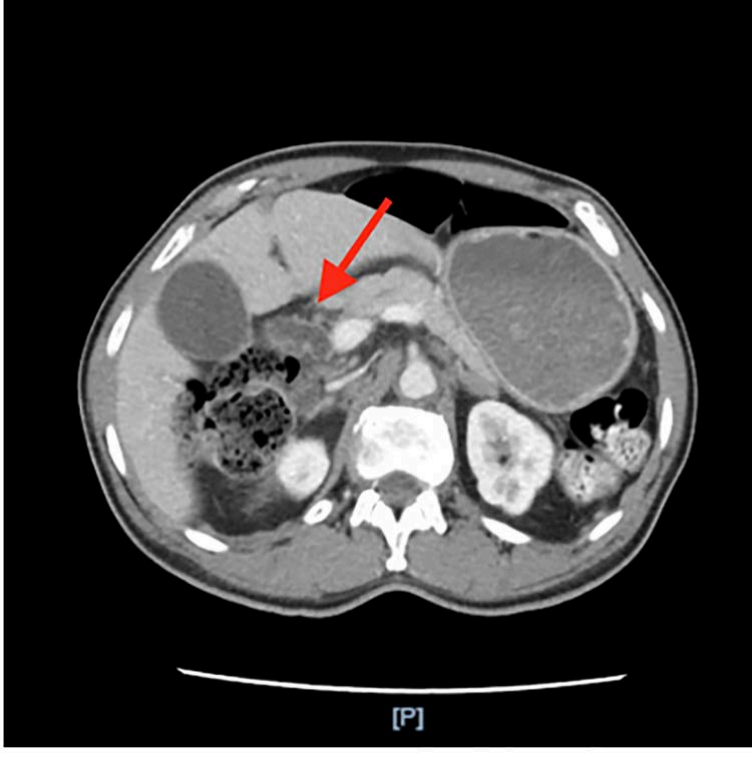
Dilated common bile duct on CT abdomen/pelvis.

**Figure 2:**
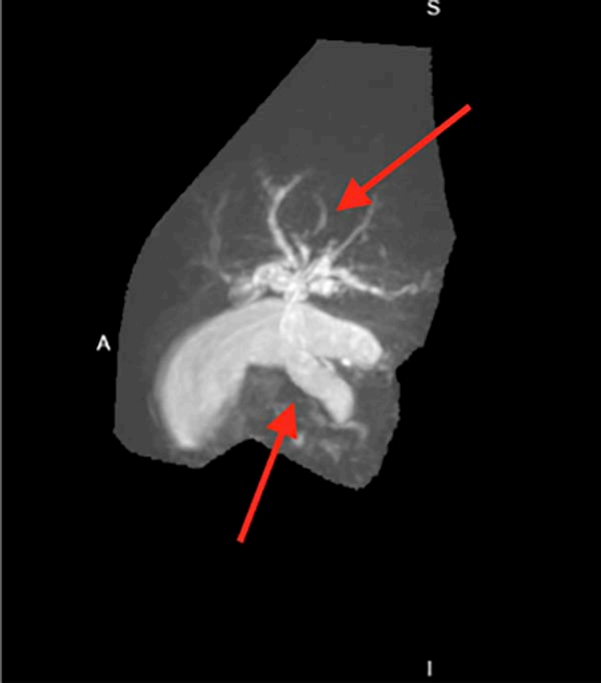
Intrahepatic and extrahepatic biliary ductal dilatation.

He had an ultrasound (US) and CT abdomen/pelvis showing abnormal intra and extra hepatic biliary ductal dilatation, suspicious for obstruction (Figs. 1 and 2). He then had an MRCP showing ductal dilation with no obvious filling defect. The total bilirubin, direct bilirubin and lipase was normal at 0.4 miligrams per deciliter (mg/dl), 0.1 mg/dl and 38 units per liter (unit/L), respectively. Liver function tests were within normal limits.

**Figure 3:**
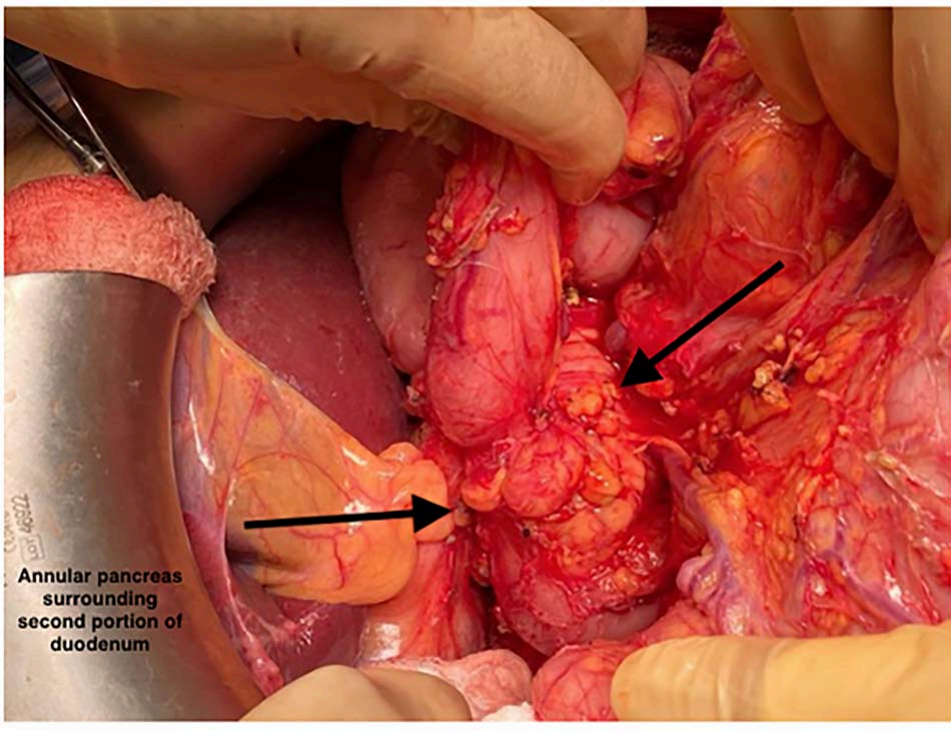
Annular pancreas encircling the pylorus and the first and section portions of the duodenum.

After the MRCP, the patient had an episode of hematemesis with a hemoglobin drop to 7.9 grams/deciliter (g/dl) from 11.9 g/dl. Gastroenterology performed an upper endoscopy and endoscopic ultrasound showing gastritis but no ulcers and severe stenosis of the pylorus which could not be traversed with an adult or pediatric scope. Extensive discussion with the radiology and gastroenterology led to a differential diagnosis of annular pancreas.

There was concern for periampullary carcinoma or cholangiocarcinoma. CA-19-9 was elevated at 42 units/ml; however, another differential for this includes biliary obstruction, which this patient had as evidenced by the intrahepatic and extrahepatic biliary ductal dilation. A marker for cholangiocarcinoma is serum Carcinoembryonic Antigen (CEA) level which was normal at 0.8 nanograms/milliliter (ng/ml) in this patient. CT triple phase and intraoperative exploration also did not suggest any malignancy.

The patient was taken to the operating room (OR) for an exploratory laparotomy and was found to have an annular pancreas encircling the pylorus and the first and second portions of the duodenum (Figure 3). No ulcers were visualized on gross examination. He was treated with a gastrojejunostomy and cholecystectomy. The patient was discharged home on postoperative day 12 while tolerating oral intake with return of bowel function and reported no further complaints at follow up.

## DISCUSSION

Despite all current diagnostic tools, preoperative diagnosis is made in only 60% of patients [7]. Preoperative diagnosis was challenging in our patient as the annular pancreas was not visible on CT scan and the patient had severe stenosis of the pylorus making it impossible to perform an ERCP. Only one recent case of a 20-year-old-female with gastric outlet obstruction secondary to annular pancreas was published. This patient had duodenal obstruction due to a stricture between the second and third portions of the duodenum resulting in the patient presenting with symptoms of gastric outlet obstruction [8]. Approximately 33% of patients with incomplete annular pancreas and 40% of patients with a complete annular pancreas have gastric outlet obstruction [9]. Our patient had stenosis of the pylorus due to the annular pancreas surrounding the pylorus or possible stricture due to previous history of gastric ulcers. Because no gastric ulcers were seen on endoscopy and patient has been on proton pump inhibitor therapy for many years, we think his symptoms were most likely due to his annular pancreas.

Our patient likely has been having postprandial abdominal pain due to gallstones for a while but did not seen medical care. In addition, this patient did not have a complete obstruction, allowing for passage of some food through the stomach and bile through the biliary tree. We think he may have delayed care for some time prior to presentation as his discomfort was only minimal.

Other recent cases of annular pancreas include a 58-year-old male patient who had annular pancreas associated with pancreatitis. He was treated with intravenous fluids and pain medications, discharged home from the ED, and has not returned for similar symptoms since [10]. A 27-year-old male presented with recurrent episodes of bilious vomiting and a dilated stomach on barium swallow, was diagnosed with a complete annular pancreas on laparotomy, and treated with a duodenoduodenostomy [11]. Last, a 40-year-old man was diagnosed with duodenal stenosis secondary to a duodenal ulcer scar due to annular pancreas surrounding the second portion of the duodenum. He was treated with a duodenoduodenostomy and had no further symptoms at follow up [12].
